# Assessment of Antimicrobial Photodynamic Therapy With Curcumin on the Shear Bond Strength of Orthodontic Bracket: An In Vitro Study

**DOI:** 10.1155/ijod/6790545

**Published:** 2025-02-25

**Authors:** Tahura Etezadi, Melika Mollaei, Parastoo Namdar, Kiana Aminmozaffari, Farhad Sobouti

**Affiliations:** ^1^Orthodontics Department, Faculty of Dentistry, Dental Research Center, Mazandaran University of Medical Sciences, Sari, Iran; ^2^Faculty of Dentistry, Dental Research Center, Student Research Committee, Mazandaran University of Medical Sciences, Sari, Iran; ^3^Dental Research Center, Student Research Committee, Mazandaran University of Medical Sciences, Sari, Iran; ^4^DSATP Candidate, Orthodontics Department, Faculty of Dentistry, University of Toronto, Toronto, Canada

**Keywords:** antimicrobial photodynamic therapy, curcumin, orthodontics, shear bond strength

## Abstract

**Background:** Antimicrobial photodynamic therapy (aPDT) is a noninvasive treatment approach that eradicates a broad variety of harmful microorganisms. This study aimed to examine the impact of aPDT with curcumin on the shear bond strength (SBS) of orthodontic bracket to enamel.

**Materials and Methods:** In this in vitro study, 45 intact human premolars were randomly divided into three groups (*N* = 15): the control group, aPDT group with curcumin 500 mg/L, and aPDT group with curcumin 1000 mg/L. After performing aPDT, the orthodontic brackets were attached to the surface of the teeth and then samples were thermocycled for 3000 cycles. The brackets were then debonded using a universal testing machine. The SBS and adhesive remnant index (ARI) were calculated. Data were analyzed in SPSS25 software. ANOVA with post hoc tests was used to compare SBS among groups.

**Results:** The findings of this study indicated that the aPDT with curcumin 500 mg/L showed the highest SBS mean, while the control group had the lowest. The average SBS difference between the control group and the curcumin 500 mg/L group as well as the curcumin 1000 mg/L group was statistically significant (*p*  < 0.05). However, the difference between the average SBS of the two curcumin groups was not statistically significant (*p* = 0.184). The experimental groups using curcumin had a considerably higher ARI value compared to the control group (*p* ≤ 0.001). However, this index was not statistically different among the C_500_ and C_1000_ groups (*p*  > 0.05).

**Conclusions:** aPDT with curcumin increases the ARI and the bond strength of the orthodontic brackets and can be used as an effective method to reduce microbial plaque and inflammation before bonding in orthodontic patients.

## 1. Introduction

Eliminating oral biofilms through brushing is challenging during orthodontic treatment due to the placement of orthodontic appliances. Therefore, the consumption of high-sugar foods and sticky substances containing sugar that might adhere to brackets and interfere with the cleaning techniques should be limited [1]. Oral mouthwashes and brushing are among the conventional cleaning approaches. However, misaligned teeth prevent complete eradication of oral pathogens, leading to poor oral hygiene in these patients [2].

Antimicrobial Photodynamic Therapy (aPDT) is a modern noninvasive strategy for decontamination of the oral cavity and deep periodontal pockets [2]. During aPDT, light exposure at a certain wavelength activates photosensitizers (PSs) or light-sensitive dyes, resulting in the production of active oxygen productions. These active cytotoxic radicals eradicate a wide range of pathogens in the target area by irreversibly damaging their membrane and DNA [3].

aPDT has shown promising outcomes as an adjunctive treatment option in inflammatory conditions such as oral lichen planus, chronic periodontitis, periodontal maintenance stage in diabetic patients, gingivitis, and gingival enlargement [4]. Moreover, this method is effective in reducing microbial load during fixed orthodontic treatment [5]. A clinical trial discovered that aPDT was more effective than full-mouth periodontal debridement in reducing the hyperplastic index score and the number of bacteria in patients with gingival enlargement caused by orthodontic treatment [6].

Curcumin is a natural PS that has widely been used in Eastern traditional medicine owing to its antioxidant, anti-inflammatory, antiseptic, antimicrobial, antiviral, and anticancer properties. Nevertheless, discoloration of resin restorations and teeth is a limitation associated with using this PS [7].

The shear bond strength (SBS) of orthodontic brackets is a crucial factor in the treatment success, which can be affected by the type of adhesive, bracket type, and surface features [8, 9]. The ideal SBS of metal brackets to tooth structure for orthodontic treatment is approximately 6–8 MPa [10, 11].

Rebonding due to bracket bonding failure is a time-consuming, costly, and risky process. Moreover, damage to the enamel surface increases during the removal of residual resin for re-bonding. Gungor et al. [12] have suggested that residual oxygen in aPDT like bleaching can disrupt the SBS of orthodontic brackets by preventing resin polymerization. Additionally, there have been reports that aPDT with methylene blue and indocyanine green reduces the SBS of orthodontic brackets [13].

Although some studies have evaluated the antimicrobial effects of aPDT with curcumin in patients with orthodontic appliance [2], its effects on the strength of orthodontic bracket bonds are controversial. Therefore, this study aimed to investigate the effect of aPDT with curcumin on the SBS of orthodontic brackets.

## 2. Materials and Methods

### 2.1. Study Design

In this in vitro study, a total of 45 healthy human premolar teeth without caries that were extracted for up to 6 months for orthodontic purposes were collected. The study protocol obtained approval from the ethics committee of Mazandaran University of Medical Sciences (IR.MAZUMS.REC.1401.14286).

Based on Sahyon et al.'s study [14], and considering a standard deviation of 8.66 and an effect size of 0.45 (80% power, 5% significance level), the total sample size was calculated as 45 (*n* = 15 for each subgroup) using the G. POWER software.

  $$\begin{array}{c} n=c\times \frac{p_{1}q_{1}+p_{2}q_{2}}{d^{2}}+\frac{2}{d}+2\approx 45. \end{array}$$

All teeth were examined under a 10x magnification stereomicroscope (SZM7045XT-B2, China). The teeth had normal anatomy and sound enamel at the buccal surface. Teeth with any enamel cracks, hypomineralization, fractures, or restorations were excluded from the study. Soft tissues surrounding all teeth were remov using a periodontal scaler. The teeth were then polished using a handpiece and rubber cap. Subsequently, the teeth were immersed in a 0.5% chloramine-T solution [7] at 3°C for 1 week for disinfection and then, stored in saline at room temperature until experiment [13].

### 2.2. Photodynamic Therapy

The samples were randomly divided into three groups (*N* = 15) using the purposive sampling method for aPDT as follows:

1. C_500_ group: aPDT with curcumin with a concentration of 500 mg/L (C_500_)
2. C_1000_ group: aPDT with curcumin with a concentration of 1000 mg/L (C_1000_)
3. Control group: No intervention applied.

The first and second groups were placed in curcumin solutions with concentrations of 500 and 1000 mg/L, respectively, in a dark environment for 5 min. Then, they were exposed to Blue LED for 4 min with a wavelength of 480 nm [14].

### 2.3. Bonding Procedure

Following the aPDT process, all samples' buccal surfaces were etched for 30 s with 37% phosphoric acid gel before being washed for 30 s with water spray. Subsequently, an oil-free air syringe was used to air-dry the surface of the teeth from a distance of 2 cm for 10 s, resulting in a chalky white enamel appearance. Next, the metal brackets (American Orthodontics, USA) were bonded to the premolar teeth. A layer of primer (Scotchbond, 3M ESPE, USA) was applied to the etched enamel and cured for 10 s. Then, the adhesive resin (Scotchbond, 3M ESPE, USA) was applied on the base of the bracket positioned at the anatomical crown's midpoint and cured by an LED device (Elipar Free light, 3M ESPE, USA) for 40 s (10 s for each side) [13].

### 2.4. Thermocycling

In preparation for thermocycling, the specimens were kept in distilled water at 37°C for 24 h. For thermocycling, the samples were exposed to 3000 cycles in a water bath at temperatures ranging from 5 to 55°C [7]. The thermocycling machine's cycles lasted 20 s each, with 10 s dwell time in the machine [13].

### 2.5. SBS Assessment

The specimens were mounted on a metallic mold using a self-curing acrylic resin (Acropass, Iran). Once the acrylic resin was set, the samples were placed in a universal testing machine (Zwick/Reoll). A force equivalent to a speed of 0.5 mm/min was applied through the machine's blade in the occlusal–gingival direction, while moving downward until debonding occurred. The maximum forces were recorded in Newtons. The conversion to SBS in MPa was calculated by dividing the recorded force by the bracket's cross-sectional area, which was accurately measured using an electronic caliper (10.4 mm^2^).

### 2.6. Adhesive Remnant Index (ARI) Assessment

**Figure 1:**
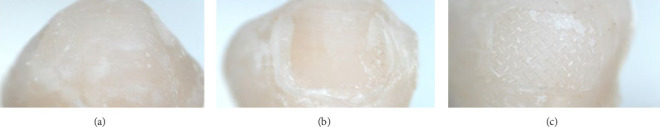
Different adhesive remnant index (ARI) values after debonding under stereomicroscope. (A) Score 0, (B) Score 1, and (C) Score 3.

Following bracket debonding, the enamel surface and bracket bases were examined under a stereomicroscope with a magnification of 10 to determine the remaining adhesive on the surface under investigation. The ARI was assessed on the enamel surface according to previous studies using the following criteria:

  Score 0: No adhesive remaining on the tooth.
  Score 1: Less than 50% of adhesive remaining on the tooth.
  Score 2: More than 50% of adhesive remaining on the tooth.
  Score 3: Complete adhesive retention on the tooth surface (Figure 1) [13].

### 2.7. Statistical Analysis

The statistical data analysis was performed using SPSS version 25. Descriptive statistics including mean, standard deviation, standard error, and minimum and maximum values were calculated for each group. One-way variance analysis (ANOVA), post hoc tests, and Kruskal–Wallis (K–W) tests were utilized to compare the SBS values among the groups. A significance level of 0.05 (*p*  < 0.05) was considered for this study.

## 3. Results

In the current in vitro study, 45 teeth were randomly divided into three groups (*N* = 15) to assess their SBS. The findings revealed a significant difference between the control group and the experimental groups, and the SBS of the orthodontic brackets in the experimental groups was higher than the control group (*p* = 0.001).

**Figure 2:**
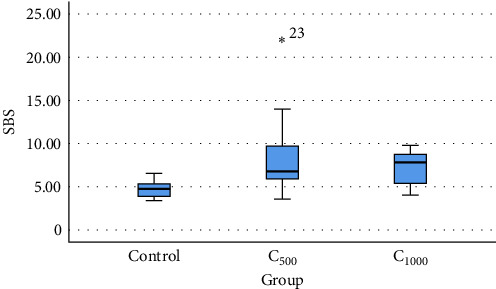
Comparing the mean shear bond strength (SBS) of the three groups.

As demonstrated in Table 1, the average SBS of all groups was equal to 6.90 ± 3.48 MPa. The amount of SBS in group A (aPDT with C_500_) was higher than in group B (aPDT with C_1000_). Moreover, the amount of SBS in the control group was lower than the other two groups (Figure 2).

The mean SBS in all three groups was different from each other, and the difference was statistically significant (*p* = 0.001). The findings from the LSD test suggested that the difference between the average SBS of the control group and the C_500_ group, as well as the C_1000_ group, was statistically significant (*p*  < 0.05). However, the difference between the average SBS of the C_500_ and C_1000_ was not statistically significant (*p* = 0.184; Table 2).

The results of the K–W test in Table 3 demonstrated that there was a statistically significant difference among the groups in terms of the ARI score. Furthermore, based on the pairwise comparisons of the group and the comparison of variables two by two, there was a significant difference between the control group and the two curcumin groups, and the ARI level in the control group was significantly lower than the experimental groups (*p* ≤ 0.001). However, there was no statistically significant difference in this variable between the C_500_ and C_1000_ groups (*p* > 0.05).  Table 4 shows the pairwise comparison among the groups in terms of their ARI score.

## 4. Discussion

Long-term orthodontic treatment can lead to complications such as root resorption, inflammation, periodontal disease, and cavities. Plaque and food particles can accumulate under the brackets and wires of orthodontic braces. When braces and orthodontic wires are present in the oral cavity, flossing can be challenging and time-consuming. Consequently, due to changes in the oral microflora, periodontal disease, cavities, and candidiasis can develop. Patients undergoing orthodontic treatment must improve their dental hygiene by using fluoride mouthwash, orthodontic brushes, different brushing techniques, and innovative technologies like aPDT [15]. In patients with fixed orthodontics, aPDT has been demonstrated to have antimicrobial and anti-inflammatory properties [2, 13, 16].

Curcumin has high absorption power in the wavelength range of 450–495 nm. The required wavelength and exposure time of the light source were determined considering curcumin's high absorption and the antimicrobial effect of light [13, 14]. Parallel to our investigation, Manso and Comeau [17] examined the impact of aPDT with curcumin and suggested that this substance could inhibit about 95% of S. mutans at the concentration of 39 µg/mL.

Failure of bracket bonding is an irreparable obstacle, which can be attributed to a variety of factors such as extended treatment duration, an inappropriate treatment plan, varying enamel quality, moisture contamination, patients' habits, and masticatory and shear stresses. For bonding efficacy, enamel restoration materials, and suitable bracket base design, the aforementioned issues must be eliminated [18]. The impact on the SBS of orthodontic brackets is one of the crucial factors to take into account when utilizing this procedure with orthodontic appliances since it might be challenging to break the connection between the bracket and the tooth [19].

The impact of aPDT on metal bracket bond strength and ARI following bonding was investigated by Baeshen [20] in 2021. Their study revealed that the bond strength can be decreased by aPDT (810 nm) with 100 mg/L of methylene blue prior to the bonding procedure [20]. Proper bond strength is crucial since an insufficient amount of it can cause bracket fracture during treatment and excessive strength can damage the enamel during the detachment procedure.

Furthermore, Abellan et al.'s [21] study compared the long-term effects of aPDT using methylene blue (0.005%) and ultrasonic scaler in fixed orthodontic patients. Their findings revealed that plaque index, gingival index, probing depth, total colony forming units (Tcfus), and inflammation levels were all significantly reduced in both groups but no significant difference was observed between these two techniques. However, following a 9-month follow-up, the aPDT group's results were more favorable. Additionally, it has been demonstrated that aPDT is a long-lasting, safe, and effective supplementary therapy for the maintenance of fixed orthodontics [21]. Nevertheless, Mirhashmi et al. [13] compared the SBS of orthodontic brackets following aPDT using 660 nm diode laser-activated methylene blue (100 μg/mL) and 808 nm diode laser-activated indocyanine green (1 mg/L). Their investigation showed that the control group had the highest SBS, and both aPDT approaches showed statistically significant SBS reductions. The primary mode of bonding to enamel or dentin is mechanical, and this is crucial for the creation of the honeycomb structure [13]. The current study's findings conflicted with the findings of this investigation. The variation in the material used as a PS may be the cause of this discrepancy.

According to Alrahlah et al. [22], curcumin and indocyanine green have the potential to be used as cavity disinfectants since they can improve the SBS of decayed dentin to composite resin. Their results indicate that curcumin has an anionic charge and that it strengthens the adhesive–dentin interaction by bonding with cationic molecules in dentin, including calcium (Ca+) and phosphate (P+). Furthermore, curcumin's hydrophobic characteristic reduced water absorption and its potential for rapid decay did not degrade SBS and created a favorable relationship with the adhesive [22]. Nevertheless, Sahyon et al. [14] investigation revealed that 500 mg of curcumin may not always provide desirable effects, yet it can provide an alternative approach as a cavity disinfectant with better SBS to decayed dentin. Furthermore, Mirhashmi et al.'s [7] study demonstrated that curcumin has an acceptable SBS in orthodontic brackets and can be used as a prebonding agent to eliminate microbial biofilms and reduce inflammation. Moreover, they reported that curcumin and riboflavin had the maximum and minimum SBS, respectively. However, the difference between the control, curcumin, and riboflavin groups was not significant [7]. Their study was the only one to investigate the effect of curcumin on the SBS of orthodontic brackets. The conflicting findings with the current study can be explained by the difference in the curcumin concentrations.

In the current investigation, the majority of curcumin samples had an ARI score of 2 and 3; while this number was significantly lower in the control group. The obtained values show that the fracture happens more in the composite. However, the maximum ARI score in studies by Mirhashmi et al. [13] and Baeshen [20] was 0, indicating tooth-adhesive failure. They used different PSs from the current research. In the other study by Mirhashmi et al. [13] which curcumin was used as a PS, the ARI scores were 1 and 2. This difference can be due to different concentrations.

Sodagar et al. [23] investigated the ARI on modified orthodontic composite containing curcumin nanoparticles. Their findings, in contrast to the current investigation, demonstrated that there was no significant difference between the experimental and control groups. However, some studies have revealed that scores higher than two are preferable. On the other hand, more adhesive on the tooth surface increases the possibility of enamel damage during polishing after bracket removal. Therefore, there have been contradictory reports on the optimal ARI score of orthodontic adhesives, and further research in this area is necessary to address these discrepancies [24–26].

There was only one previous study assessing the impact of curcumin on the SBS of orthodontic brackets. Using different concentrations of curcumin was one of the strengths of the current research. However, the in vitro nature of this investigation cannot stimulate the accurate condition in the oral cavity. Further investigations can be conducted on different PSs, bonding systems, and brackets material, to provide comprehensive knowledge for orthodontists.

## 5. Conclusions

The current investigation showed that aPDT with curcumin significantly increases SBS in comparison to the control group. Using aPDT with curcumin 500 mg/L results in the highest amount of SBS in orthodontic brackets. Nevertheless, the difference between the use of 500 mg and 1000 mg/L concentration in terms of change in the SBS was not significant.

## Figures and Tables

**Table 1:** Descriptive data about the three groups of control, C_500_, and C_1000_.

SBS	*N*	Mean	Standard deviation	Standard error	Minimum	Maximum
Control group	15	4.759	0.907	0.234	3.43	6.55
C_500_	15	7.8	2.97	0.76	3.57	23.22
C_1000_	15	7.208	2.03	0.525	4.03	9.78
Total	45	6.61	2.49	0.37	3.43	13.97

Abbreviation: SBS, shear bond strength.

**Table 2:** The mean difference of shear bond strength (SBS) in three groups of control, C_500_, and C_1000_.

Groups		Mean difference	Standard error	*p* value
Control group	C_500_	−3.126	0.783	0.001
	C_1000_	−2.448	0.783	0.003

C_500_	Control Group	3.126	0.783	≤0.001
	C_1000_	0.678	0.783	0.391

C_1000_	Control Group	2.448	0.783	0.030
	C_500_	−1.545	0.783	0.391

**Table 3:** The percentage and frequency of adhesive remnant index (ARI) among the three groups of control, C_500_, and C_1000_.

Group		0	1	2	3	Total
Control group	*N*	2	4	9	0	15
	% ARI	100%	66.7%	45%	0%	33.3%

C_500_	*N*	0	1	6	8	15
	% ARI	0%	16.7%	30%	47.1%	33.3%

C_1000_	*N*	0	1	5	9	15
	% ARI	0%	16.7%	25%	25.9%	33.3%

Total	*N*	2	6	20	17	45
	% ARI	100%	100%	100%	100%	100%

**Table 4:** The pairwise comparison of the adhesive remnant index (ARI) score among the groups.

First–second groups	Test statistic	Standard error	Standard test statistic	*p* value	Adj. sig.*⁣*^*∗*^
Control—C_500_	−14.733	4.438	−3.320	0.001	0.003
Control—C_1000_	−15.967	4.438	−3.598	≤0.001	0.001
C_500_-C_1000_	−1.233	4.438	0.278	0.781	1.000

*⁣* ^*∗*^Significant values were adjusted by Bonferroni correction for multiple testing.

## Data Availability

The data would be provided by contacting the corresponding author upon reasonable request.
